# A 3D Printer Guide for the Development and Application of Electrochemical Cells and Devices

**DOI:** 10.3389/fchem.2021.684256

**Published:** 2021-07-02

**Authors:** Ana Luisa Silva, Gabriel Maia da Silva Salvador, Sílvia V. F. Castro, Nakédia M. F. Carvalho, Rodrigo A. A. Munoz

**Affiliations:** ^1^ Grupo de Catálise Ambiental e Sustentabilidade Energética, Instituto de Química, Departamento de Química Geral e Inorgânica, Universidade do Estado do Rio de Janeiro, Maracanã, Rio de Janeiro, Brazil; ^2^ Núcleo de Pesquisa em Eletroanalítica, Instituto de Química, Universidade Federal de Uberlândia, Uberlândia, Brazil

**Keywords:** 3D printing, electrochemical cells, electrochemical devices, electroanalyisis, additive manufacturing

## Abstract

3D printing is a type of additive manufacturing (AM), a technology that is on the rise and works by building parts in three dimensions by the deposit of raw material layer upon layer. In this review, we explore the use of 3D printers to prototype electrochemical cells and devices for various applications within chemistry. Recent publications reporting the use of Fused Deposition Modelling (fused deposition modeling®) technique will be mostly covered, besides papers about the application of other different types of 3D printing, highlighting the advances in the technology for promising applications in the near future. Different from the previous reviews in the area that focused on 3D printing for electrochemical applications, this review also aims to disseminate the benefits of using 3D printers for research at different levels as well as to guide researchers who want to start using this technology in their research laboratories. Moreover, we show the different designs already explored by different research groups illustrating the myriad of possibilities enabled by 3D printing.

## Introduction

We are experiencing the fourth industrial revolution, better known as Industry 4.0. The production modes and management of the industries have changed deeply in the last decade. The accelerated progress was driven by the research and development (R&D) of cleaner technologies, with smarter, faster, cheaper, and higher-quality manufacturing processes (Almada-Lobo, 2016). In this context, the emerging technology of 3D printing has stood out in the last decade and has promised to revolutionize the production in several sectors, such as R&D, aerospace, industry, diagnostics, healthcare, dentistry, engineering, civil construction, education, food, arts, among others. (Huang et al., 2013; Kanada, 2014; Siebert and Teizer, 2014; Wong and Pfahnl, 2014; Gupta et al., 2015; Micallef, 2015; Pallottino et al., 2016; Iyer et al., 2017; Derossi et al., 2018; Hamilton et al., 2018; Jones and Spencer, 2018; Prakash et al., 2018; Javaid and Haleem, 2020; Zhu et al., 2021)

3D printing is defined as an additive manufacturing (AM) technology created to build three-dimensional objects. Accordingly, AM is a process that produces 3D objects (hollow or filled) by the deposit of the raw material layers by layers. (Bell and Bell, 2014) The most popular example of AM is masonry: the construction of structures using units connected or not, like brick walls. Some pieces can be built by removing the raw material, such as carving an object out of wood, this technique is known as subtractive manufacturing. Many machines, such as lathes and machining centers, use computer numeric control (CNC) to perform the subtractive manufacturing processes (Minhat et al., 2009; Ertekin et al., 2013).

Although 3D print technology has only gained notoriety more recently, it dates from the early 1980s when Hideo Kodama first described something similar to what came to be the stereolithography (SLA) technique years later. In 1984, Chuck Hull, founder of 3D Systems, developed the first rapid prototyping machine (3D printer) and the first patent was granted in 1986. Two years later, Carl Deckard launched another technology, selective laser sintering (SLS), and in the following year, 1989, Steven Scott Crump, co-founder of Strasys, developed the technology for fused deposition modeling (FDM) printers. The term FDM was patented by Strasys and because of that, a new open source nomenclature was created to refer to the printers that use fused filaments as raw material: fused filament fabrication (FFF) (Tully and Meloni, 2020).

Among the infinite possibilities of application in Chemistry, one of the research areas that has been highly benefitted by 3D printing advances is electrochemistry. Research groups in electrochemistry and/or electroanalysis are currently looking for standardized and customized ways to produce electrochemical devices that are more efficient, low-cost, sustainable, and more durable. Among the main objects of study are the electrodes (anode, cathodes, sensors, biosensors, and immunosensors) (Silva et al., 2015; Duarte et al., 2017; Honeychurch et al., 2018; Silva et al., 2018; Cardoso et al., 2019; Cardoso et al., 2020a; Rocha et al., 2020), electrochemical cells (Raju and Basha, 2005; Zhakeyev et al., 2017; Cardoso et al., 2018; Katseli et al., 2020; Lim et al., 2020), microfluidic systems (Duarte et al., 2017; Rossi et al., 2018), among others (Zhu et al., 2016; Cardoso et al., 2020b), especially with regard to the applications for energy storage (batteries and supercapacitors) (Zhang et al., 2020), water splitting (Ambrosi and Pumera, 2016; Zambiazi et al., 2020), hydrogen evolution (Iffelsberger et al., 2020), fuel cells (Bian et al., 2018) and sensors (Cardoso et al., 2020b). Faced with this challenge, 3D printing has been surprisingly useful to prepare an unlimited sort of customized electrochemical devices.

**SCHEME 1:**
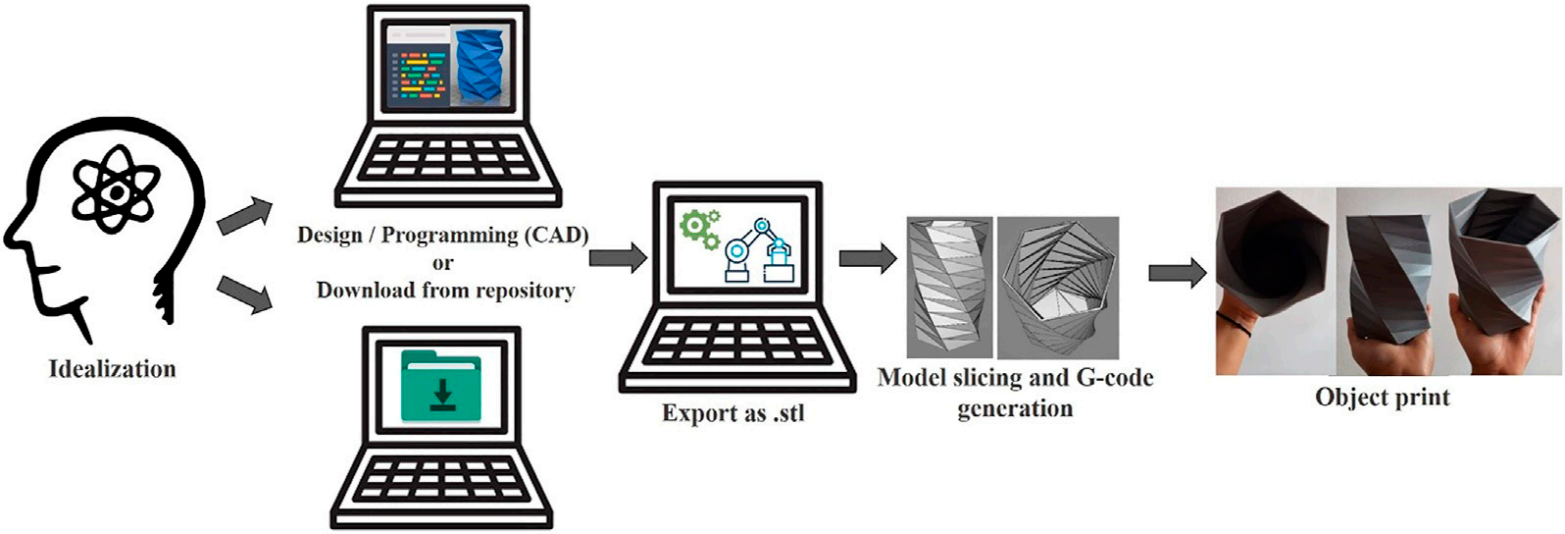
Flowchart of the main stages of the 3D printing process.

Even with this brief historical evolution of 3D printing, some issues still need to be addressed. In the course of this review, we will explain in detail the basics necessary to start printing in 3D and the main steps involved in the entire process are presented in Scheme 1. The first question to be addressed is what is needed to print 3D objects. Unfortunately, 3D printers are not as easy to use as inkjet printers. It is necessary to take into account several factors such as application, choice of equipment, raw material, and level of knowledge of the operator. Next, the main advantages and disadvantages of 3D printing technologies will be highlighted, and finally, its use in electrochemical applications with a special focus on the development of electrochemical cells will be presented. In addition, we will introduce the new trends about 3D printers and how they can contribute to the research and development process for the area of chemistry. Furthermore, the types of 3D printers the available raw materials, will be presented herein while the software options to create, configure and convert 3D parts into a command to the printer to do the job, will be presented in detail in the Supplementary Information SI

## Types of 3D Printer

**FIGURE 1:**
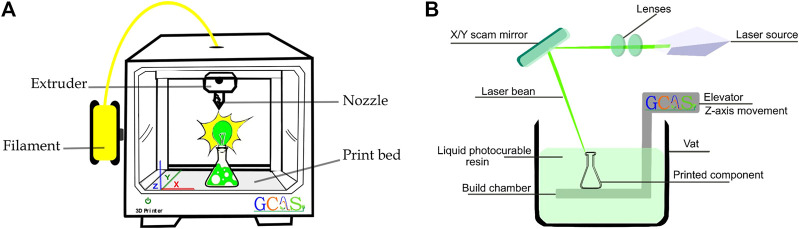
Schematic diagram of: **(A)** Cartesian FFF and **(B)** SLA 3D printers (Source: Produced by the author).

We will start this journey by presenting the several types of 3D printers that are currently available on the market: FDM or FFF, SLA, SLS, Digital light processing (DLP), Multi Jet Fusion (MJF), PolyJet Direct Metal Laser Sintering (DMLS), Electron Beam Melting (EBM), among others. The main parts of the 3D printers of the FFF and SLA type are shown in Figure 1.

The most widespread 3D printing technique is Cartesian FFF, which uses a thermoplastic filament as raw material to build 3D objects. As detailed in the next lines, Cartesian FFF printers can be opened or closed, with direct drive or bowden, with fixed or mobile bed, but they all have some features in common that define their classification, which goes beyond the type of filaments used as raw material. These printers have three axes (*X*, *Y* and *Z*) that guide the position of the object in the print bed. The movement of the *X*, *Y* and *Z* axes may change according to the printer manufacturer, but they will always follow the Cartesian coordinate system. These movements can also be performed by the table when moving back and forth, or up and down, as well as through the extruder. An extruder, in turn, is present in every FFF printer, and it is the part itself responsible to build the 3D objects. It is further divided into two parts: a cold tip that pulls in the rigid filament, and a hot piece where the filament is fused until it is fluid enough to escape through the nozzle and be deposited on the print bed. The print bed can be hot or cold, however, most 3D printers manufactured nowadays use hot beds on their machines. The main advantage of the hot bed is to enable the use of filaments that need a hot surface to adhere to the layers on top of each other. (Wang et al., 2017; Rodríguez-Panes et al., 2018; Cardoso et al., 2020b)

Finally, enclosing the main common parts of an FFF printer are the nozzles. There are several types of nozzles, with different orifice sizes ranging from 0.1 to 1.0 mm and made of different materials. They are directly related to two very important printing parameters: the printing speed and the resolution of the built objects. In summary, the smaller the nozzle orifice, the faster the printing can be and the better the finished piece will be. The most common material used for the manufacture of the nozzle is brass, but other materials can also be used as plated copper, hardened steel, stainless steel, among others.

The second most popular 3D printing technology, better known as Vat polymerization (VP), uses a photopolymerizable resin consisting of monomers and oligomers, a crosslinking agent, and a photo indicator in a vat as a printing raw material. (Tully and Meloni, 2020; Carrasco-Correa et al., 2021) In this case, the resin is cured or solidified when a light source selectively contacts it. There are currently three VP technologies: SLA, digital light process (DLP), and LED-LCD or MSLA.

SLA 3D printer uses an ultraviolet light laser with a beam opening between 150 and 300 μm that scans the surface of the photoactivated resin vat. A dense three-dimensional object is formed through the movement of two galvanometer mirrors: one on the *X*-axis and the other on the *Y*-axis; in this way the beam only irradiates the region to be solidified. To reduce the printing time of the SLA 3D printers, DLP 3D printing technology was created. While SLA uses an ultraviolet laser, DLP uses a digital projector to generate a single image of each layer at once through a DMD and a set of lenses that project the image over the resin.

The latest 3D printing technology is LED-LCD, although it was created as an evolution of the DLP 3D printers, they compete more with SLA 3D printers. LED-LCD 3D printers have a similar working principle to DLP, but they use a LED matrix as a light source and a LCD device instead of DMD. LCD devices, in addition to block or let the light in, also manage to filter it by the variation of the light intensity independently on each pixel and improves the quality of the printed surface to be as good as SLA prints.

As mentioned before, there are several types of 3D printers that use different raw materials such as polymer powder, metal powder, glass, ceramic, plaster, among others. Hereafter we will explain succinctly about the SLS, direct metal laser sintering (DMLS), multi-jet modeling (MJF), and electron beam melting (EBM) technologies, as these are more often used in industries, although their features are very interesting for research inside and outside the academy.

The SLS and DMLS technologies have the same operating principle, the difference is in the raw material used to produce the pieces: SLS 3D printers use polymer powder and DMLS 3D printers use aluminum or titanium powder. During the printing of the objects, a thin layer of dust is dispersed on a platform inside the build chamber, then the printer heats the powder to a temperature slightly below the melting point of the raw material and a laser beam scans the specific area of the powder thus drawing the prototyped object. This laser scan occurs in a cross section of the 3D model, mechanically fusing the particles until creating one single solid piece. After printing the parts, the build chamber cools quickly to guarantee the appropriate mechanical properties and prevent structural deformations. After reaching room temperature, the piece can be removed from the build chamber. An advantage of this technique is that, unlike the FFF and VP technologies, you can reuse the leftover dust of the print because their properties are preserved. (All3 dp, 2021; Formlabs, 2021)

3D printers of the EBM type work very similarly to DMLS ones, the difference is in the heat source used to sinter the metallic powder. These printers use an electron beam that is produced by a cannon of Tungsten filament under vacuum. The cannon projects the electron beam in an accelerated way over the metallic powder layer deposited in the build chamber, fusing them selectively until the object is formed. MJF technology is the latest example of 3D printers in this guide, they work by selective application of the fusing agent to a thin layer of nylon powder using an inkjet matrix. The nylon powder is melted layer by layer until the object is finally formed. Afterward, all the remaining dust is removed by an integrated vacuum system that also removes residual dust from the printed object. Finally, the pieces are dyed in a previously chosen color, usually black.

In summary, we present in Table 1 the main advantages and disadvantages of the FFF and VP 3D printers.

**TABLE 1:** Advantages and disadvantages of the FFF and VP 3D printers.

Advantages/ Disadvantages	FFF	Vat polymerization
Low cost of printers	Yes	No
Wide variety of materials available at affordable prices	Yes	No
Possibility to make changes to the extruder	Yes	No
Prints large volume objects	Yes	No
Possibility of transparent printing	Yes	Yes
Possibility to print multiple materials simultaneously	Yes	No
Required post-processing	No	Yes
Easy maintenance	Yes	Yes
Low organic solvent compatibility	Yes	Yes
Z and X-Y resolution	No	Yes

After this brief overview of the operation of the different types of 3D printing, we will present the raw materials used by each technology in more detail, focusing now on FFF and VP 3D printers.

## Raw Materials for 3D Printer

**SCHEME 2:**
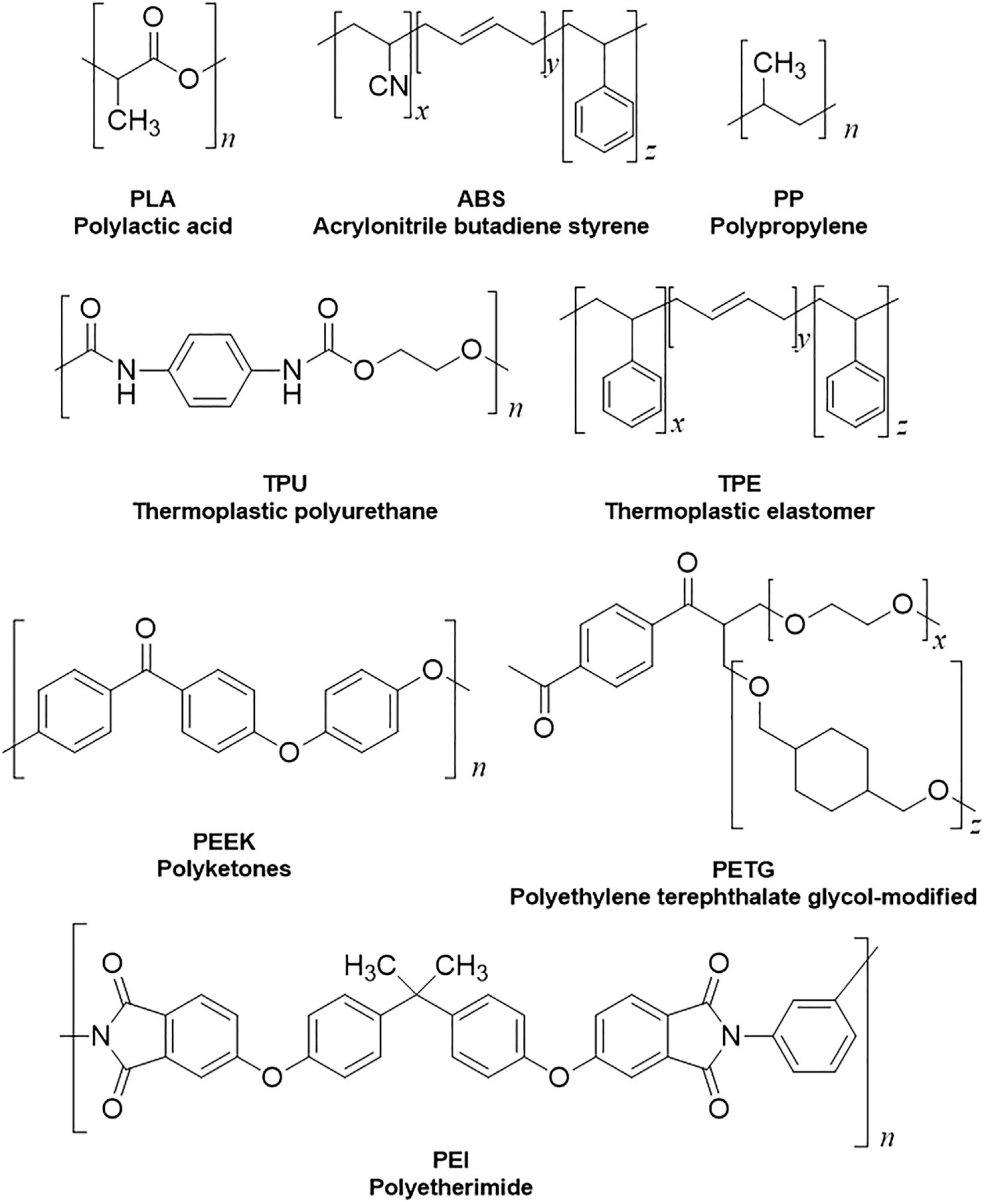
Chemical structures of the polymers used as raw materials for 3D printing.

After choosing the most suitable technology for the desired application, the other factor to be considered is the raw material to be used, for instance, filaments and resins are the most employed for open-source 3D printers. The search for materials that meet the needs of different areas has strengthened the collaborations between research groups, also strengthening the relationship between academia and industry and, consequently, accelerating the development of new filaments and resins. The filaments, for example, have very different physical, chemical, and mechanical properties according to the characteristics of their polymer precursor. (Zaharia et al., 2017; Gümperlein et al., 2018; Singh et al., 2020; Carrasco-Correa et al., 2021) Although there are hundreds of filaments on the market, the most used and/or the most promising for R&D we will be highlighted: Polylactic Acid (PLA), Acrylonitrile Butadiene Styrene (ABS), Polyethylene Terephthalate Glycol-modified (PETG), Thermoplastic Polyurethane/Thermoplastic Elastomer (TPU/TPE), Polypropylene (PP), Taulman Tritan High Tensile Polyester (Tritan), Polyketones (PEEK), Polyether Ketone Ketone (PEKK) and Polyether Imide (PEI). The chemical structures of the polymers are represented in Scheme 2.

PLA is a filament easy to handle, so it is the most suitable for beginner operators. The versatility of PLA is closely related to one of its main advantages, its biodegradability. On the other hand, PLA has low thermal and mechanical resistance, it is also very hygroscopic, which can make the piece potentially fragile, and when used in the wrong way it can bring some problems such as nozzle clogging. The printing process is faster using PLA because of the relatively lower printing temperatures from 180 to 230°C.

The second most used filament is ABS, it has high impact resistance, good thermal resistance, and moderate flexibility. ABS has a fast contraction that causes warping in the parts, making the final object in most cases useless. To reduce these problems, it is recommended to use a closed printer to avoid air current in the object during the printing, however, the printer should stay in an airy place as ABS releases toxic vapors when heated. ABS filament printing temperature is higher than that of PLA, varying from 210 to 250°C, which increases the printing time, as the printer takes longer to heat up. PLA and ABS are the most employed raw materials for the construction of electrochemical cells and their composites in combination with conductive agents for sensors. (Cardoso et al., 2020b)

**FIGURE 2:**
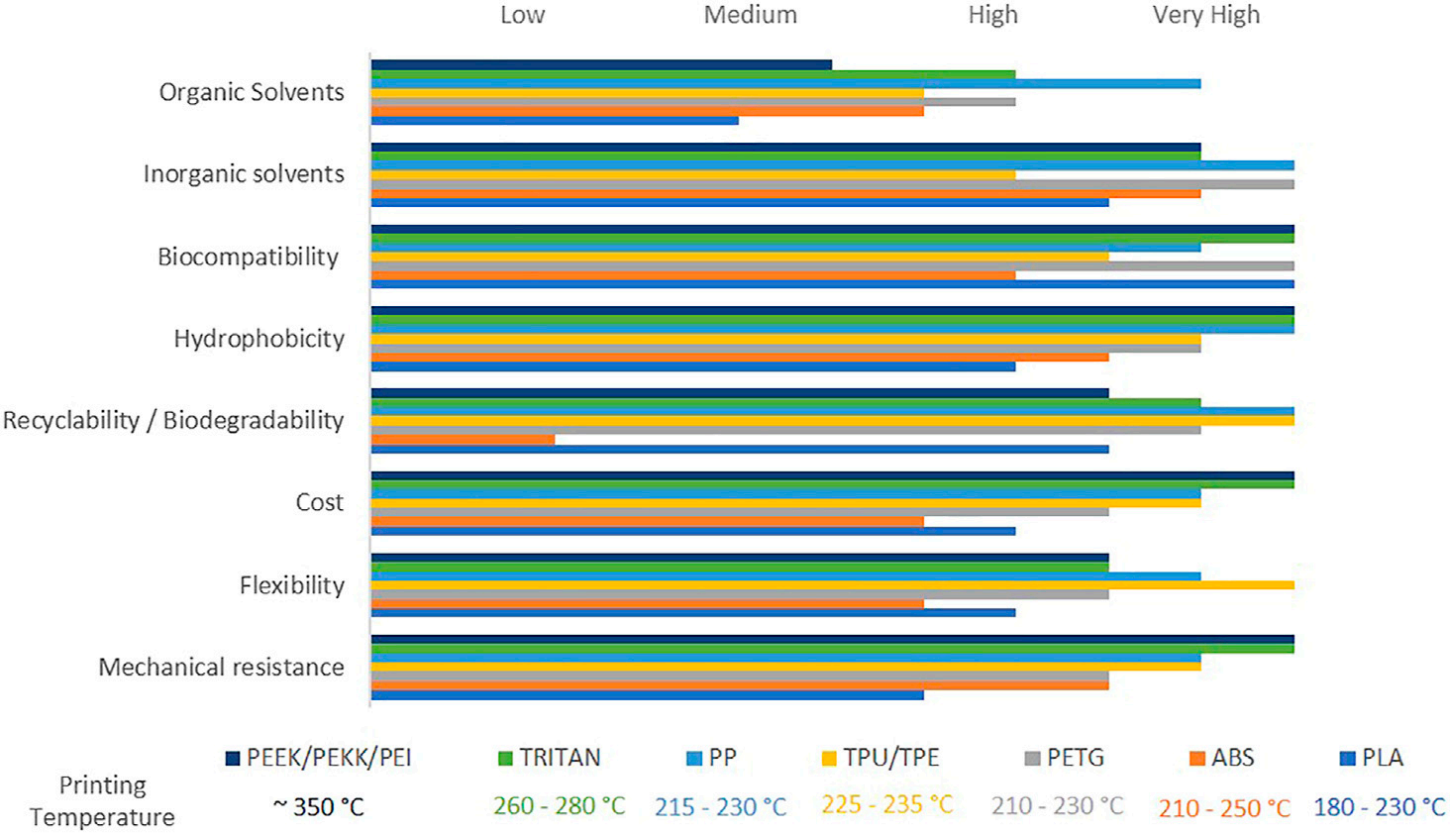
Graphical representation of features of the raw polymeric materials used for FFF 3D printing (Source: Produced by the author from the data in Erokhin et al., 2019; Ligon et al., 2017; Appropedia.org; and the fabricants Rosemount Analytical Inc. and Curbell plastics).

Other filaments not so well explored for electrochemical applications but still with notable advantages are PETG, TPU/TPE, PP, Tritan, PEEK, and PEI. Figure 2 shows some features of the raw materials, such as cost, recyclability, biodegradability, flexibility, hydrophobicity, biocompatibility, and printing temperature. When the 3D printed devices combine such characteristics (biodegradability/flexibility for instance), promising practicable sensors for measurements in non-aqueous media can be built.

PETG is a recyclable material approved by FDA and has printing temperature ranges between 210 and 230°C, but a very fine adjustment is necessary to set the optimum printing temperature, which makes it a difficult material for beginners to handle. However, their advantages are worth it, it has high mechanical and impact resistance, it is flexible, hydrophobic, biocompatible, and does not deform easily. Because PETG is considered safe for food, its main application is in the use of household items and food containers. PP has good elasticity, high resistance to fatigue and chemical resistance, and is very durable. To print using PP, the temperature of the extruder must be between 215 and 230°C. TPU, on the other hand, has higher elasticity and flexibility than PP, has very high abrasion resistance, and is also very resistant to impacts. Like PETG, it is not easy to handle. Its extrusion temperature ranges from 225 to 235°C.

To keep up with the evolution of 3D printing technology, several high-performance filaments have been created. Tritan, PEEK, PEKK, and PEI are filaments of very high mechanical and thermal resistance. They are quite light and relatively easy to print. In addition, Tritan is an FDA approved material and has a print temperature range of 260–280°C, making it a viable option for use on bench 3D printers. Unlike PEEK, PEKK, and PEI, these filaments require high extrusion temperatures around 350°C. The data summarized in Figure 2 show that the filaments are mostly chemically resistant to inorganic solutions. However, the same does not apply to chemical resistance to organic solvents. The highest chemical resistance to organic solvents was shown by PP followed by PETG and Tritan. (Erokhin et al., 2019; Appropedia 2021)

As well as in the case of filaments, choosing the right resin for a particular application is essential. Resins are preferred by those who wish to create objects with higher resolution of details, with a smooth, dense, or less porous surface, besides the ease of finishing after printing. In addition, time is a determining factor in the choice of printers that use resins instead of filaments. Generally speaking, they are divided into two groups: standard resins and advanced resins. Standard resins are the most common, they have excellent cost benefit, they offer good mechanical properties, they are translucent and can be dyed in many different colors, and their most usual application is in the print of prototypes for various purposes. Some resins present similar properties to the filaments, as is the case of zABS that resembles the ABS filament. The group of advanced resins is for more specific applications and it is widely used in engineering, dentistry, medicine, games, and jewelry. Within this category are biological-based resins, transparent, water washable, flexible, biodegradable, castable, fluorescent, and fast curing. Within their specificities, they have better mechanical resistance, better print resolution, low odor, higher precision, moisture resistance, scratch resistance, among others.

An important note is the color of the filaments and resins must be taken into account for each application, as the dye used in the manufacture of the raw material can interfere with the product's properties. For example, if the purpose is to develop a composite electrode based on PLA and graphite and you use a white filament (the white color is usually made from TiO_2_). All factors must be taken into account to ensure that the efficiency of this electrode is only that of its precursors without taking into account the color of the PLA filament. For instance, TiO_2_ within the filament may provide photocatalytic porperties to the final 3D-printed device (Browne et al., 2020).

## Examples of 3D Printer Applications in Electrochemical Devices

### 3D Printed Electrochemical Cells for Sensing and Other Applications

In this section, we will present a collection of publications that addresses the use of 3D printers in the development of electrochemical devices. Since 2012 there has been an exponential growth in articles dealing with 3D printing technology related to electrochemistry, as reported in previous works of made critical reviews highlighting the development of electrochemical sensors using this technology (Cardoso et al., 2020b; Hamzah et al., 2018). 3D printing technology has diversified and increased the possibilities to develop and produce specific electrochemical cells for several applications. As mentioned before, many publications have addressed the use of this technology in electrochemistry and electroanalytical methods, but little has been reported on the development of electrochemical cells. Here we will present examples of electrochemical cells built from a 3D printer and their respective applications. Table 2 summarizes a myriad of cell designs according to the application envisioned by the different research groups.

**TABLE 2:** **-** 3D printed electrochemical cells, including microfluidic devices and wearable platforms, their respective printing technique, polymers, and applications.

Electrochemical device	3D printing technique	Polymer (body or substrate)	Electrodes	Design (scheme and/or real image)	Post-Treatment/Electrode activation	Application	Ref.
**Electrochemical cells and devices produced with a single 3D printer**							
Flow cell for sensing (Line A)	SLA	Liquid acrylate resin	Boron-doped diamond or gold working electrode and quasi-reference electrode (Ag/AgCl wire)	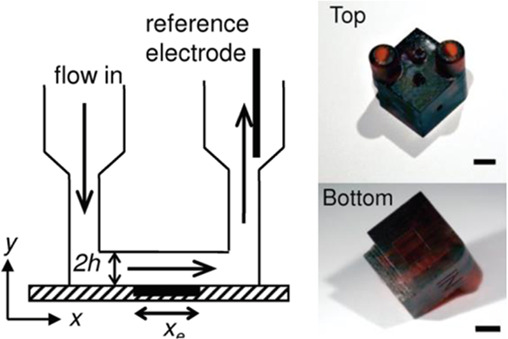	Cleaned with isopropyl alcohol, acetone, and finally rinsed thoroughly with distilled water. Then UV-light curation for 30 min.	Microchannels (length of 3.5 mm, width of 3 mm and height of 0.2-0.25 mm) with a boron-doped diamond electrode embedded and an external quasi-reference electrode placed at the outlet. Experiments were performed with the redox probe ferrocenylmethyl	Snowden et al. (2010)
						Trimethylammonium.	
Flow cell for electrolysis (Line B)	FFF	ABS	Ni foil (as cathode and anode)	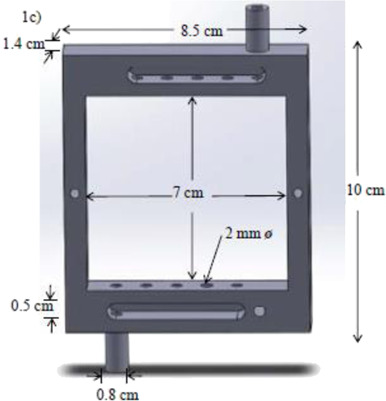	Not required	Flow cell prototype for electrodeposition, corrosion, metal ion removal, organic oxidation and fuel cell	Ponce de Leon et al. (2014)
Flow cell for gas sensing (Line C)	SLA	Liquid acrylate resin	Au-plated stainless-steel thread rod	Dimensions of the closed box-shaped cell (5 cm x 3 cm x 3 cm) with solution inlet and outlet at opposite sides and insertion of the three electrodes	Not required	Hydrogen gas detection in metals under flow conditions	Schimo et al. (2015)
BIA cell for sensing (Line D)	FFF	ABS	Screen-printed electrodes	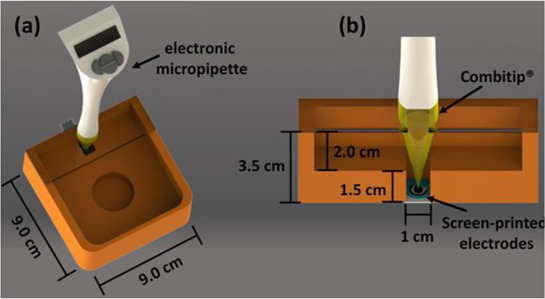	Not required	Paper enzymatic reactor for indirect glucose sensing based on the amperometric detection of H_2_O_2_ on s screen-printed carbon modified with Prussian blue within the 3D printed BIA (electronic pipette for injections)	Dias et al. (2016)
Assembling BIA cell for sensing (Line E)	FFF	ABS	Screen-printed carbon, graphite, gold and 3D-printed carbon	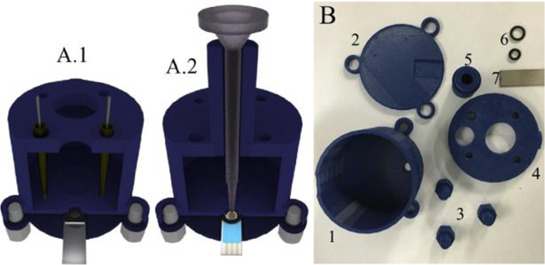	Not required	Amperometric sensing of dopamine, catechol, tert-butylhydroquinone, diclofenac, and dipyrone using different working electrodes	Cardoso et al. (2018)
					Polishing of the 3D-printed electrode		
Mechanized analytical platform with autosampler and a wall-jet cell (Line F)	FFF	ABS	Screen-printed electrodes	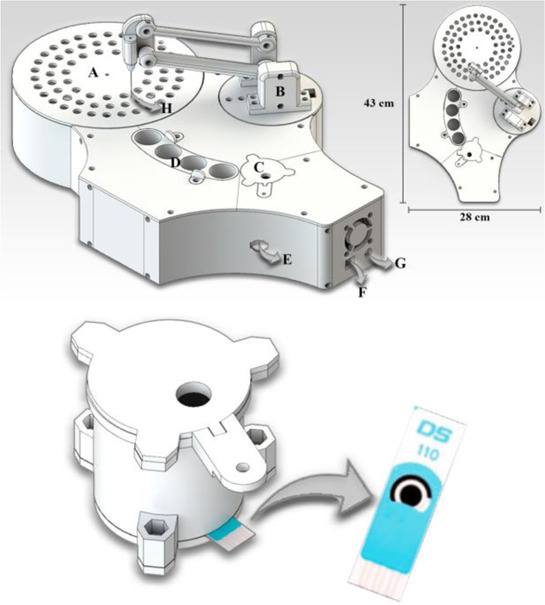	Not required	Autosampler controlled by Arduino for sample injection (500 nL) using a syringe micropump from several reservoirs constructed over the 3D printed platform. Injections were made over the working electrode of screen-printed system placed in a wall-jet configuration for amperometric detection (paracetamol, antioxidants and cocaine detection)	Mendonça et al. (2019)
Assembling wall-jet flow cell for HPLC (Line G)	FFF (cell) and FFF (electrodes)	ABS (body of the cell) and conductive PLA (electrodes)	3D-printed CB/PLA	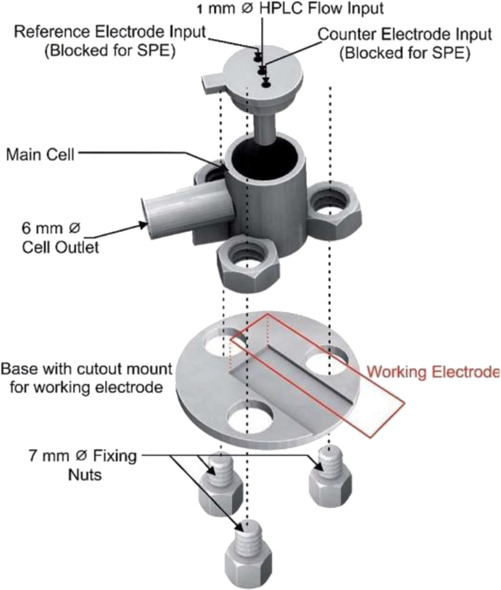	Electrochemical activation of the 3D-printed electrodes	Electrochemical flow cell with 3D-printed carbon electrodes for coupling via PTFE tubing to an HPLC system for the amperometric determination of NBOMes (illicit drugs) after chromatographic separation	Elbardisy et al. (2020)
			Graphene/PLA, graphite/PLA, graphite sheets and screen-printed electrodes				
Assembling cell for sensing (Line H)	FFF (single step fabrication)	ABS (body of the cell) and conductive PLA (electrodes)	3D-printed PLA with carbon black electrodes	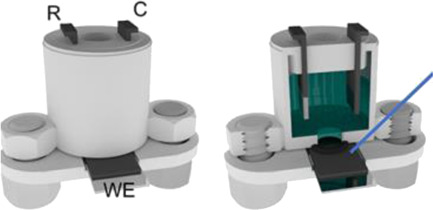	Electrochemical treatment of the 3D-printed electrodes	Fabrication of a complete additively manufactured electrochemical cell applied for the voltammetric detection of different analytes of biological interest (dopamine, uric acid and ascorbic acid)	Richter et al. (2019)
Assembling cell combining sampler and sensor(Line I)	FFF	ABS (body of the cell) and graphene- PLA as sampler and working electrode	3D-printed graphene-PLA working electrode	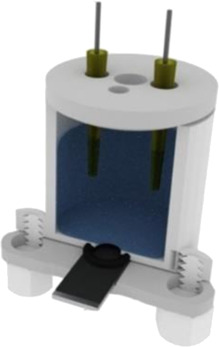	Mechanical polishing of the electrodes	The working 3D-printed electrode served as a collector (swab) of either explosive or gunshot residues at the forensic scene and as a voltammetric sensor of the collected molecules, TNT in explosives and Pb and Sb in gunshot residues	Cardoso et al. (2019) and Castro et al. (2020)
Assembling cell for sensing and biosensing (Line J)	FFF	PLA (body of the cell) and conductive PLA (electrodes)	3D-printed PLA with graphene rod-shaped electrodes	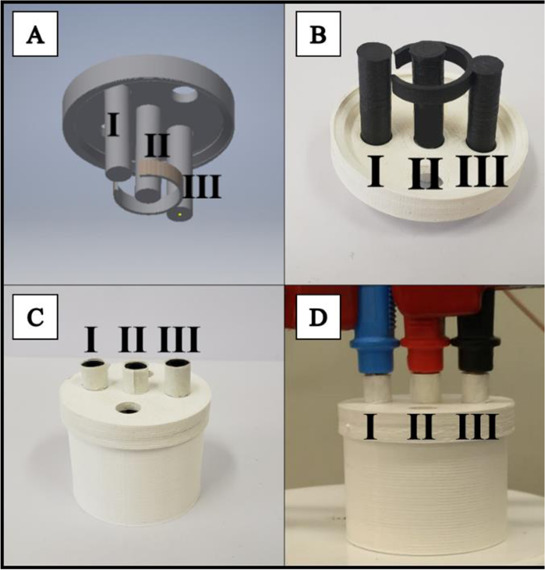	Chemical treatment with nitric acid and borohydride	Three-electrode system 3D-printed in a single step using conductive filament connected to insulating 3D-printed support for electric connection. The working electrode was treated to generated reduce graphene oxide for further immobilization of enzymes for biosensing of catechol and serotonin	Silva et al. (2020)
Cell with separated reservoirs for the electrodes (Line K)	FFF	Natural polyamide 12	3D-printed PLA-CNT electrodes	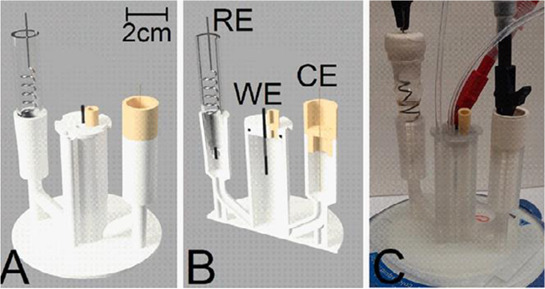	Not required	Investigation of the electrochemical oxidation of hydrazine coupled with CO_2_ reaction. The 3D-printed cell contains separated compartments for the three electrodes and enables reactants to be introduced and inspected under oxygen-free conditions. The central compartment (21 mm inner diameter x 66 mm height) accomodates the working electrode (WE) and allows gas inlet.	Escobar et al. (2020)
Cell with the three electrodes embedded for sensing (Line L)	FFF (dual 3D printer for single step fabrication)	PLA (body of the cell) and conductive PLA (electrodes)	3D-printed carbon black-PLA working electrode	3D-printed box-shaped cell (1.6 cm x 1.6 cm x 1 cm height) with the three electrodes embedded with inner volume of ∼2 mL	Electrochemical treatment of the 3D-printed electrodes	Mercury determination by anodic stripping voltammetric after gold film formation over the 3D-printed PLA with carbon black	Katseli et al. (2020)
Lab-in-a-syringe voltammetric cell (Line M)	FFF	PLA (body of the cell) and conductive PLA (electrodes)	Graphite paste modified electrode	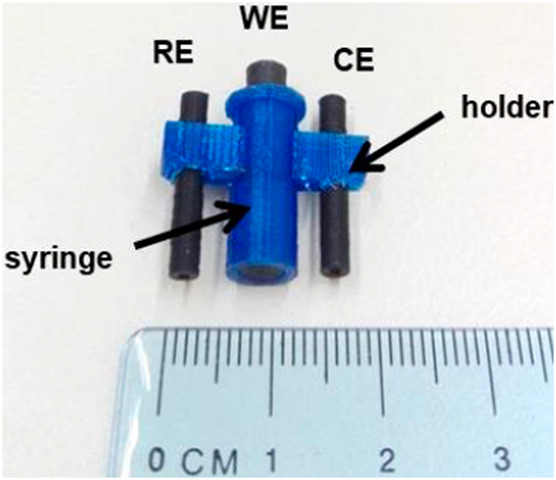	Graphite paste modified with metallic-organic framework	Metal (mercury or lead) preconcentration through a 3D-printed syringe for further anodic stripping voltammetric determination. The three-electrode system is placed within a 3D-printed hollow cylinde cell	Kokkinos et al. (2020), Vlachou et al. (2020)
**Electrochemical devices fabricated by combining 3D printers with other 3D printing tools**							
Assembling cell for sensing (Line N)	FFF and inkjet printing	ABS (body of the cell) and conductive PLA (electrodes) or Ag ink	Ink-jet printed Ag electrode; 3D printed ABS-carbon as counter; Ag/AgCl wire/ KCl (agar)/3D printed junction as reference	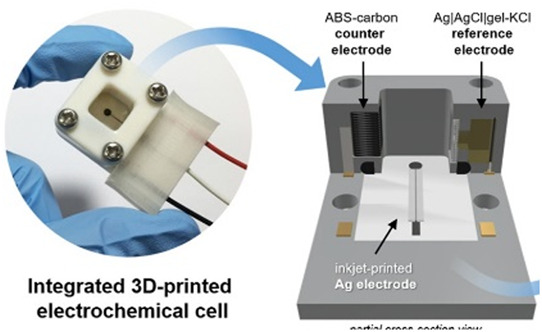	Electrochemical treatment of ink-jet printed Ag working electrode	Ink-jet printed electrode combined with FFF 3D-printed cell containing 3D-printed counter electrode and 3D-printed junction of the reference electrode. Nitrate determination in aqueous samples by voltammetric detection through the electrochemical reduction of nitrate ions.	Sibug-Torres et al. (2021)
Ready-to-use device (Line O)	FFF and 3D pen	PLA (template of the device) and conductive carbon PLA (electrodes)	3D-printed graphene-PLA working, counter and reference electrodes	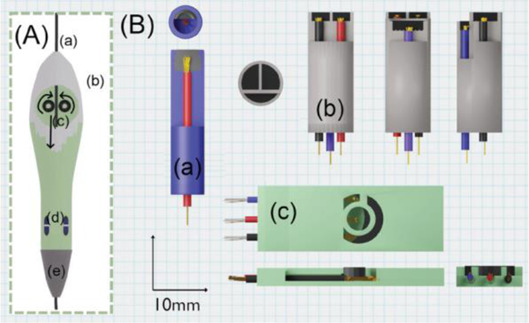	Chemical treatment of the 3D-printed graphene-PLA electrode by immersion in DMF	The fabricated devices were demonstrated for the determination of dopamine, metals and hydrogen peroxide after surface modification with Prussian blue.	Cardoso et al. (2020a)
Ready-to-use device (Line P)	SLA and 3D pen	Acrylic resin (template of the device) and conductive carbon black PLA (electrodes)	3D-printed graphene-PLA working, counter and reference electrodes	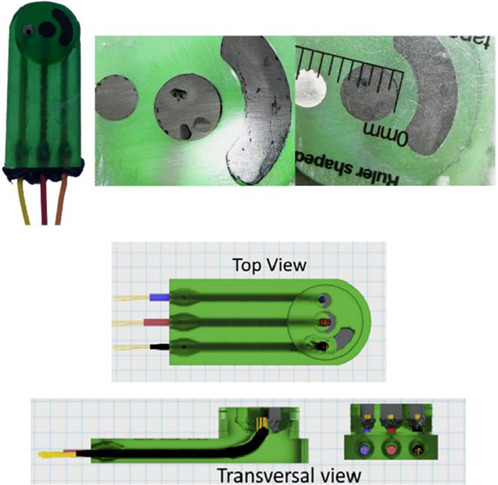	Electrochemical treatment of carbon black PLA electrode; AgCl formation on 3D printed carbon reference electrode	Templates were fabricated using SLA (30 mm x 15 mm) while a 3D pen was used to print the electrodes. Reference electrode modified with Ag/AgCl provided more stable responses. The device was applied for the analysis of a single drop solution containing the explosive TNT	Cardoso et al. (2020c)
Assembling cell for sensing (Line Q)	FFF and 3D pen	PLA (body of the cell) and conductive PLA (electrodes)	3D-printed carbon black-PLA counter and reference electrodes (II c and II d in A) using the 3D pen and planar working electrodes	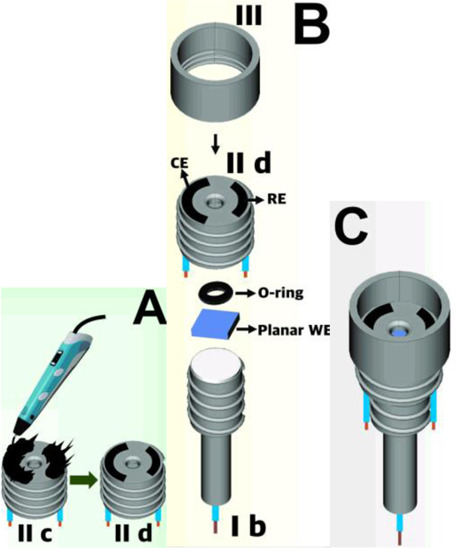	Electrochemical activation of the 3D-printed carbon black electrode as well as of the boron-doped diamond electrode	The combination of FFF 3D-printed cell (cylinder of 26 mm diameter and 15 mm height) with a 3D pen to fabricate counter and reference electrodes within the cell. Different planar working electrodes can be assembled. Connections performed by electric copper wires inserted through the bottom. Applied for determination of 17α-ethinylestradiol in a contraceptive pill as well as other drugs.	Ferreira et al. (2021)
**Microfluidic electrochemical devices**							
Microfluidic cell with electrode embedded (Line R)	FFF	PLA (body of the microfluidic cell) and graphene- PLA (electrodes)	3D-printed graphene-PLA electrodes	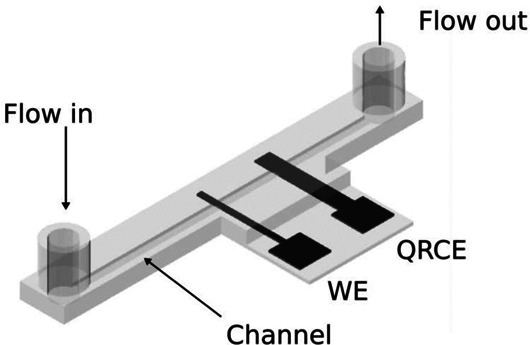	Gold film electrodeposition	Single-step fabrication of a microfluidic device (two-electrode system with a working and counter/reference electrodes) for the amperometric detection of catechol	O’Neil et al. (2019)
Fluidic cell coupled to a SIA system (Line S)	FFF	PLA (body of the cell) and conductive PLA (electrodes)	3D-printed graphene-PLA working electrode	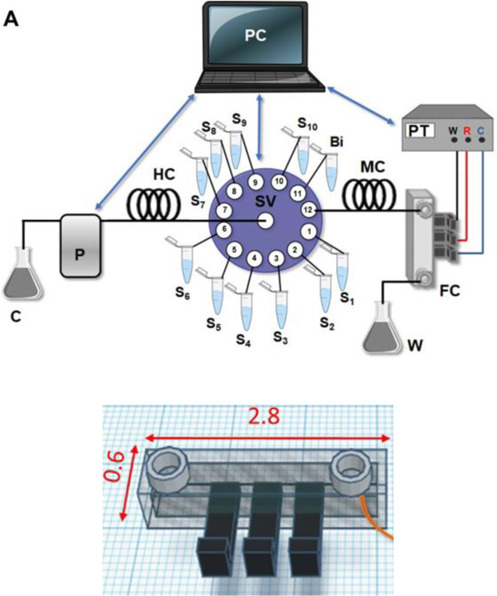	Bi electrodeposition on the 3D-printed working electrode	3D-printed fluidic cell using PLA (zoom out at the bottom and dimensions in cm) combined with a sequential injection analysis (SIA) system connected to a pump to inject sample, standard and modifier solutions. The proposed system was applied for the determination of cadmium and lead in honey after sample digestion.	Baltima et al. (2021)
Microfluidic cell with potentiometric sensor embedded (Line T)	FFF	PLA	Screen-printed silver electrode modified with sulfide	3D-printed chip device containing a microfluidic channels for mixing sample and electrolyte before potentiometric detection close to the solution outlet. The device has three solution inlets. Electrodes are screen-printed on the chip	Silver screen-printed modified with sulfide	3D-printed microfluid device that enables solution mixing (derivatization step) integrated with a potentiometric sensor based on Ag_2_S (two-electrode system with working and reference electrodes) for the selective sulfide determination	Pol et al. (2019)
Thread-based microfluidic device (Line U)	FFF	ABS (body of the cell) and conductive PLA (electrodes)	3D-printed PLA with carbon black electrodes	The three electrodes are 3D printed on a ABS platform at which the cotton thread is placed over the three electrodes working as solution carrier due to the capillary action	Electrochemical activation of the electrodes	3D-printed platform containing the three-electrode system at the top and a cotton thread aligned between inlet and out reservoirs to serve as a microfluidic channel without the need of a pump. The micro-FIA system was applied for nitrite determination in well waters	Carvalho et al. (2021)
Microfluidic cell with pencil graphite electrode integrated (Line V)	SLA	Liquid acrylate resin	Pencil graphite electrodes	Transparent 3D-printed device with electrodes inter-connected (distance between the electrode of 0.6 mm and graphite thickness of 0.5 mm)	Electrochemical activation of the pencil graphite electrodes	Flow injection determination of clozapine using graphite electrodes embedded in a 3D printed microfluidic device using cotton threads to produce the microflow sensing platform for real-time measurement of antipsychotic clozapine level	Senel and Alackkar (2021)
Microfluidic cell with macro or microelectrodes embedded (Line W)	PJM (Objet Connex 350 Multi-material 3D printer)	Liquid acrylate resin (VeroClear)	Platinum Black microelectrodes embedded within a PEEK cylinder and Nafion-coated glassy carbon	Transparent microfluidic (0.50 x 0.50 mm) platform with two electrodes placed along the channel	Channel cleaning with compressed air, then with polyimide-coated capillaries, compressed nitrogen and sonication.	This is the first device employing microelectrodes along the 3D printed microfluidic channel. Applied for sensing dopamine, NO and oxygen.	Erkal et al. (2014)
**Wearable electrochemical devices**							
Wearable sensors							
Wearable sensor with integrate electronics (Line X)	FFF	Not mentioned	Screen-printed electrodes	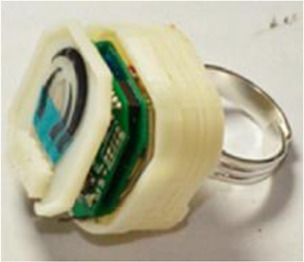	Not required	Ring-based screen-printed sensor for vapor detection of DNT, H_2_O_2_ (explosive derivatives) and organophosphate nerve agent. Electronics for measurement are integrated within a 3D-printed box	Sempionatto et al. (2017)
Wearable ring sensor (Line Y)	FFF	Polyurethane (ring) and conductive PLA (electrodes)	3D-printed graphene-PLA working electrode	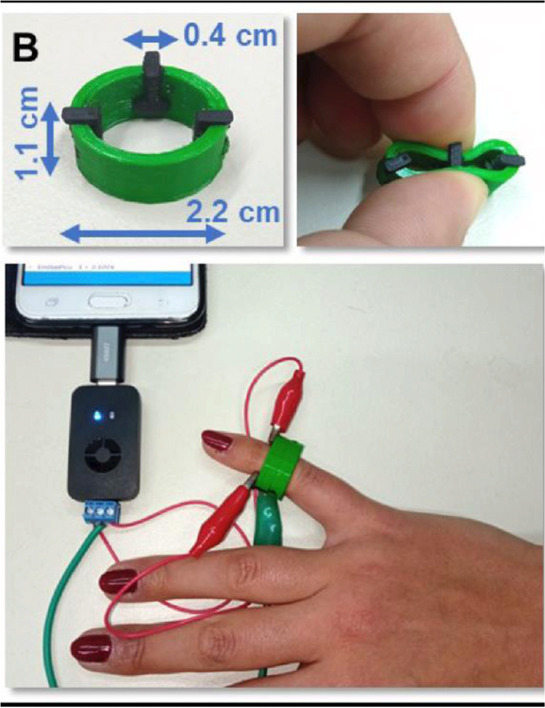	Gold electrodeposition	Enzymeless glucose sensing in sweat on the carbon black/PLA electrode modified with gold embedded in the 3D-printed ring device using chronoamperometry. Glucose sensing of volunteers before and after meal (1 h and 2h)	Kateseli et al. (2021)
Wearable device containing a sensor (Line Z)	FFF	ABS	Flexible thermal-printed graphite electrodes	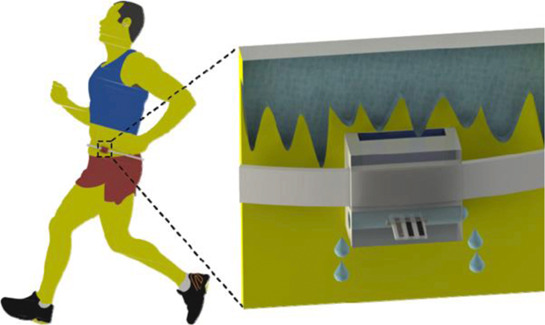	Bismuth film electrodeposition	3D-printed wearable device containing the electrochemical device was fixed at the body using an elastic tape for zinc determination in sweat	Dias et al. (2019)

Images reproduced with permission from American Chemical Society (Snowden et al., 2010; Richter et al., 2019; Sempionatto et al., 2017; Katseli et al., 2021; Dias et al., 2019), Italian Association of Chemical Engineering (Ponce de Leon et al., 2014), Elsevier (Dias et al., 2016; Cardoso et al., 2018; Mendonça et al., 2019; Cardoso et al., 2019; Silva et al., 2020; Escobar et al., 2020; Sibug-Torres et al., 2021; Cardoso et al., 2020c; Ferreira et al., 2021; O’Neil et al., 2019; Baltima et al., 2021); Brazilian Chemical Society (Cardoso et al., 2020a), Royal Society of Chemistry (Elbardisy et al., 2020) and Multidisciplinary Digital Publishing Institute (Vlachou et al., 2020).

According to a search carried out on the Web of Science® database using the keywords “3D printing” and “electrochemical cells”, we can highlight some pioneer works using SLA and FFF 3D printing techniques on different occasions. Previously to SLA and FFF, we can mention a simple 3D-printed electrochemical cell fabricated using a acetoxy silicone polymer cured at room temperature for 12 h that enabled voltammetric measurements and highlighted the potential of 3D printing (Symes et al., 2012). SLA has been investigated earlier than FFF and the pioneering work reported by Snowden and coauthors (Snowden et al., 2010) presented an electrochemical (U-shaped) flow cell in which the working electrode (diamond doped electrode) was placed at the bottom of the cell in a thin-layer configuration, while the counter-reference electrode (Ag/AgCl wire) was inserted through the outlet channel. The total process of fabrication, 3D printing and post-treatment, took 6–8 h for a device of 10 cm height (shown in Table 2, line A), was time-consuming, and employed a liquid resin composed of an acrylic oligomer, dipentaerythritol pentaacrylate, propoxylated trimethylolpropane triacrylate, photoinitiator, and stabilizers. The resolution is much higher than the FFF 3D-printed devices, however, longer time of fabrication, the need for post-treatment and curation, and the use of toxic resins are some drawbacks that make SLA less popular than FFF. Nevertheless, one of the first works on the development of functional electrochemical cells by FFF 3D printer is from 2014. On that occasion, Ponce de Leon et al. (2014) used two 3D printers powered with ABS to build an indivisible flow cell using a relatively large size (electrode area 49 cm^2^). A printer UP!2 Plus (by PP3DP) was used to make the electrolyte channel and a printer Ultra® 3SP (by Envision TEC) was used to make the endplates. Then, it was characterized using a conventional K_4_Fe(CN)_6_/K_3_Fe(CN)_6_ redox probe to analyze mass transport and compare with other flow cells manufactured via traditional machining (Ponce De Leon et al., 2014). The authors proposed the use of this 3D-printed cell to investigate electrodeposition, corrosion, and electrochemical remediation (metal removal or advanced oxidation processes) as well as fuel cells (illustrated in Table 2, line B).

Two different 3D technologies were used to build chemiluminescence flow-cells: using a MJM 3D printer (3D System) and using a CNC milling (Datron). The cells were constructed with particularities, the first with a single detection zone and the second with two separate detection zones. Both were compared to conventional systems using flow-injection analysis (FIA) and HPLC with fast chemiluminescence reactions (Spilstead et al., 2014). A flow microcell was developed using a SLA 3D printer to measure the electrochemical loading of hydrogen *in situ* via cyclic voltammetry using Kelvin probe measurements (Schimo et al., 2015), which is probably one of the first works using 3D printer technologies for the development of electrochemical cells. In 2016, the development of paper-based enzymatic reactors for glucose detection using a 3D printed batch injection analysis (BIA) cell was described. BIA is a portable analytical system that provides fast analyses when combined with amperometric detectors as it employs an electronic micropipette that precisely controls volume and injection rate, which are fundamental features that affect the current response (Rocha et al., 2018). The BIA cell (inner volume of 100 ml) had a low production cost ($5) and was built in 4 h using a FFF 3D printer with ABS both from Prusa Movtech (MovtecH Comercial Tecnologia LTDA-ME) (Dias et al., 2016). A screen-printed electrode modified with Prussian blue (also known as artificial peroxidase), selective to the amperometric detection of H_2_O_2_ (product generated at the enzymatic reactors), was placed inside the cell and an electronic micropipette was assembled over the BIA cell to control the injection rate and volume of the standard and sample solutions. Glucose was enzymatically converted by the H_2_O_2_ detected at the electrode, enabling indirect glucose sensing. Another work worth mentioning is from Cardoso et al. (2018), who developed a multiuse flow cell used in the amperometric detection of several drugs both via FIA and BIA using several working electrodes, including 3D printed electrodes (Cardoso et al., 2018). The advantage of this electrochemical cell is the possibility of using either steady-state or flow conditions (FIA and BIA) as well as several types of working electrodes, with a total volume of electrolyte of 80 ml. The FDM 3D-printed parts of the cell were fabricated within 6 h using ABS filament, but PLA filament can also be employed to construct this multiuse electrochemical cell. Miniaturized counter and reference electrodes are placed through the top cover of the cell and a rubber O-ring was placed between the working electrode and a metallic plate used for electric contact. Hence, these 3D-printed electrochemical cells require other parts that were not 3D-printed. The same research group reported a portable, mechanized, and fully 3D-printed platform that comprises an autosampler, injection syringe pump, and electrochemical cell (Mendonça et al., 2019). This platform (43 × 28 × 15 cm) can manipulate microvolumes (0.5 µl) from a sample tray containing 68 vials and inject over a screen-printed electrode placed inside a 3D-printed electrochemical cell (inner volume from 50 to 10 ml) using a 3D-printed syringe pump. This report shows a potential application of 3D printing for the construction of mechanized analytical systems. All these electrochemical cells are presented sequentially in Table 2 (line C to F).

The previous examples of electrochemical cells fabricated by 3D printing were designed to allow the insertion of conventional (working, counter, and reference) electrodes or more often miniaturized electrodes. Considering the potential of 3D printers to fabricate electrodes, the literature shows examples of complete 3D-printed electrochemical cells containing the three-electrode system also fabricated by 3D printing. To reach this goal, conductive filaments for FFF 3D printers are required and there are some commercially available sources, either based on PLA or ABS. The conductive element is a carbon-based material, and the manufacturers report the presence of carbon, graphene, or carbon black particles within the polymeric matrix. It is also possible the lab-made production of conductive filaments and a section on a recent review by Cardoso et al. (2020b) is devoted to electrochemical sensors enabled by 3D printing.

The BIA systems using inverted working electrodes have been inspired on electrochemical cells developed for HPLC system, hyphenated at the end of the chromatographic column. A 3D-printed electrochemical designed for HPLC was reported (Elbardisy et al., 2020) and shown in Table 2 line G. This electrochemical cell containing the three-electrode system assembled was applied in the determination of the illicit drug named as NBOMes (different chemical structures) using the amperometric detection. Different working electrodes were evaluated, including 3D-printed carbon-PLA electrodes, however, screen-printed electrodes showed the highest analytical responses.

A complete 3D-printed electrochemical cell was reported by Richter et al., 2019 (Table 2 – line H) (Richter et al., 2019). The three electrodes were printed using conductive PLA containing carbon black as rectangular plates (3 cm × 1.5 cm) with a thickness of 0.75 mm, while the reference was modified with Ag/AgCl to increase the stability of the potential application. Counter and reference electrodes are assembled through the cover that presents perfect holes to their insertion, while the working electrode is placed at the bottom of the cell and pressed by 3D-printed screws. The cell compartment (cover, bottom, screws, and body) was printed in ABS (inner volume of 5 ml). All parts of the cell were printed using a direct drive extruder FFF 3D printer. Other electrochemical cells with very similar designs (Table 2 – line I) were proposed by the same research group (Cardoso et al., 2019; Castro et al., 2020) in which all parts of the cell are 3D-printed in a row, except the electrodes. The working electrodes were 3D-printed using graphene-PLA filaments, and one interesting application is their use as a sampler (swab) of gunshot or explosive residues. The counter and reference electrodes used in both cases were miniaturized platinum and Ag/AgCl/KCl, respectively. One disadvantage of these types of cells is the need for assembly before use; however, the advantage is the possibility of reuse after water cleaning, with minimal contamination between analysis; moreover, this cell can be printed using a simpler FFF 3D printer without the need of a dual extruder 3D printer. Many other designs can be evaluated depending on the electrochemical application.

A 3D-printed electrochemical cell designed to easily connect the electrodes with cables from a potentiostat was proposed (Silva et al., 2020) as shown in Table 2 – line J (A – image of the electrode; B – 3D printed electrodes; C – 3D-printed cell; D – assembled cell with connectors). This cell works with a larger volume than the previous version, but the electric connection of the electrodes can be considered more robust. The working electrode within this cell was proposed as a biosensor to detect biomolecules in biological fluid and water samples.

Another design was explored to fabricate electrochemical cells for experiments using conventional electrodes and gas inlet, such as the report by Escobar et al. (2020) (Escobar et al., 2020). The authors investigated the chemical reaction between CO_2_ and hydrazine using an FFF 3D printed cell (Table 2 – line K: images A and B are schemes while image C presents the real image), whose design presents a configuration with a separated compartment for each electrode interconnected by a fluidic channel. The gas inlet occurs through the central container (capacity of 20 ml) at which the working electrode is placed, and the gas outlet occurs through a side compartment at which the counter electrode is placed.

Katseli and coauthors reported the fabrication of a complete electrochemical cell applied for Hg(II) determination using a dual extruder FFF 3D printer, in which two commercial filaments were used: non-conductive PLA and conductive PLA containing 22% wt. carbon black from Proto Pasta® (Katseli et al., 2020). The electrochemical cell was box-shaped (2 × 2 × 1 cm, described in Table 2 – line L) and the three electrodes were printed using the conductive filament over the bottom of the cell, crossing the sidewall to establish the electric connection. The inner volume of the cell was 2–3 ml and the determination of Hg was greatly improved after the addition of a gold plating solution. One criticism about the reported design that needs to be considered is the settling of the working electrode at the central position of the cell between the counter and reference electrodes. Another issue is the need for a gold film formed by electrodeposition before the analysis, which brings an additional step and cost. To overcome this drawback, the same research group proposed another electrochemical cell, in which the working electrode can be removed from the cell to be applied for preconcentrating mercury before the voltammetric analysis (Kokkinos et al., 2020). The great advantage of this device is the single-step fabrication of a ready-to-use electrochemical device, with no need for assembling before use (Table 2 – line M).

The combination of two 3D printing techniques has been proposed to fabricate electrochemical devices and some examples are highlighted in a separated section in Table 2. FFF and ink-jet printing were used to construct a 3D-printed electrochemical device for the determination of nitrate in water. FFF was used to fabricate the cell in ABS and the counter electrode using carbon-ABS. The reference electrode was Ag/AgCl/KCl in agar with a 3D-printed junction. The working electrode was an ink-jet printed Ag on a flexible substrate, electrochemically treated before nitrate determination. The complete assembled system is compact, as shown in Table 2 – line N (highlighted the integrated cell, scheme with different parts, and the ink-jet printed Ag working electrode) (Sibug-Torres et al., 2021).

The combination of FFF 3D printers with 3D pen to fabricate electrochemical sensing devices has been demonstrated. A handheld 3D pen can be used to construct tiny electrodes within 3D-printed platforms, which enabled the design of multiple electrochemical devices (Table 2 – lines O to Q). The first two examples are from electrochemical devices similar to commercially available screen-printed electrodes using either FFF or SLA to fabricate a customized template for further coverage with the conductive carbon filaments using a 3D pen (Cardoso et al., 2020a, and Cardoso et al., 2020c). A real image of the device constructed using SLA is presented in Table 2 (Cardoso et al., 2020c). Another example was recently reported employing FFF and 3D pen, in which an assembling cell was fabricated by FFF and the 3D pen was used to apply the conductive carbon filament over the created template to serve as counter and reference electrodes (Ferreira et al., 2021). Different planar working electrodes were evaluated for drug screening analysis. Table 2 illustrates how the device is assembled and in which positions the three electrodes are placed within the cell.

The use of 3D pen to fabricate on demand miniaturized electrodes on 3D printed platforms provides great promises for the development of novel microfluid devices as well as lab-on-a-chip systems.

### 3D Printed Microfluidic Electrochemical Devices

Due to the freedom of design, the 3D printing of microfluidic electrochemical devices has been envisaged and this section is devoted to exemplifying some potential applications. Table 2 also presents a second separated section to illustrate some of these devices. 3D printing enabled the development of microfluidic channels with electrodes inserted along the main channel by different strategies. Using a dual extruder FFF 3D printer, O’Neil et al., 2019 reported the fabrication of a microfluid flow electrochemical system in a single step (a single 50 mm long channel with a cross-sectional dimension of 1.5 × 1.0 mm^2^), using non-conductive PLA and conductive PLA containing graphene (from Black Magic®) as shown in Table 2 – line R. The device comprised a hydrodynamic fluidic channel printed using non-conductive PLA, containing a reservoir at both end sides of the system for flow in and out. Two electrodes were printed along the channel using the conductive filament (the first one served as working electrode and the second as pseudo-reference or counter electrode). The system enabled a flow rate between 0.5 and 3.5 ml min^−1^. This work demonstrated one of many other possibilities to fabricate microfluidic devices with electrodes embedded after a single-step fabrication.

Baltima and coauthors proposed a 3D-printed microfluidic cell to be combined with a sequential injection analysis (SIA) system with the three electrodes embedded along the channel (Baltima et al., 2021) as shown in Table 2 – line S. This fluidic device was fabricated by FFF using PLA in a similar design described by O’Neil et al., 2019. The combination of the 3D-printed fluidic devices with the SIA system provides a great improvement on sample throughput and can be automated. The automated sequence of analysis involves filling with supporting electrolyte, aspiration of the sample or standard solutions, aspiration of Bi(III) solution for *in situ* film formation with a flow rate between 1.8 and 0.6 ml min^−1^. The system was stopped for the anodic stripping voltammetric analysis and then cleaned underflow for the next analysis.

Another strategy to fabricate microfluidic devices (Table 2 – line T) was reported by Pol et al. (2019), which combined 3D printing with screen-printing technology to produce a platform for sulfide potentiometric detection (Pol et al., 2019). Using an FFF 3D printer, a microfluidic platform was fabricated containing two parallel channels, one of them with a mixer for sample preparation (two solution inlets) and the second for supporting electrolyte insertion (one inlet). These channels are connected close to the outlet solution at which both electrodes (working and reference) are positioned. The reference and working electrodes were prepared by screen-printing an Ag/AgCl and Ag/Ag_2_S inks, respectively.

A thread-based microfluidic device placed within a 3D-printed platform was proposed by Carvalho et al. (2021) (Table 2 – line U). The cotton threads worked as the microfluidic channels responsible to carry the solution from the injection point to the electrochemical detection, named as micro-FIA. The 3D printed platform contained a three-electrode system (all of them were 3D printed using a conductive PLA containing carbon black) as well as the support to stand the threads to perform the microfluidic determination of nitrite (as a proof-of-concept). This pioneering platform shows another potential application of 3D printing towards the development of microfluidic electrochemical devices.

Another design of a microfluidic device for FIA was proposed using SLA and the three graphite electrodes inserted within the cell (Senel and Alachkar, 2021). The authors used pencil graphite electrodes, which were electrochemically treated and then applied for clozapine determination (Table 2 – line V).

Most of the examples shown so far were obtained by FFF 3D printing. The VP techniques (including SLA) have enormous potential for the fabrication of microfluidic devices but lack investigation on the combination of this 3D printing technique with electrochemical detectors, probably due to the low resolution of the printed parts (Waheed et al., 2016). One interesting example of a microfluidic device with integrated and reusable electrodes was reported by Erkal et al. (2014), who used high-resolution poly jet modeling (PJM) and an acrylate-based resin (Erkal et al., 2014). This 3D printing technique enabled the fabrication of a single 500 × 500 µm channel platform, working with a flow rate of 6 μL min^−1^, which allows the integration of conventional working macroelectrodes as well as microelectrodes (encapsulated within epoxy) for bioanalytical applications, when low sample volumes are accessible (Table 2 – line W).

### 3D Printed Wearable Sensors

Additive manufacturing has been a powerful tool to make feasible novel wearable sensors, which can be considered adaptative electrochemical cells able to monitor chemical species in biological fluids in real-time. This section will show a few examples of creative systems developed by 3D printing and illustrated in Table 2. One of the first examples of a 3D printed device that can be used as a ring was reported by Joseph Wang`s group (Sempionatto et al., 2017). The authors reported a 3D-printed device containing integrated electronics and a screen-printed electrode (covered with semi-solid agarose-based electrolyte) assembled to a ring with Bluetooth communication to a computer. The electrochemical device was evaluated for the detection of 2,4-dinitrotoluene (DNT), which is a contaminant commonly associated with the explosive TNT and H_2_O_2_ (degradation product of peroxide-based explosives) in liquid and vapor phases (Table 2 – line X).

Following the concept of a ring-shaped device, Katseli reported a 3D-printed wearable device for glucose sensing (Katseli et al., 2020). The difference from the previous example is that the electrodes were also 3D-printed within the 3D printed ring, so it is a complete 3D printed wearable sensor (Table 2 – line Y). Glucose detection was possible after gold electrodeposition on the 3D-printed carbon black/PLA electrode for the electrocatalytic oxidation of glucose, detected on sweat before and after a meal. The device is flexible and ready to use for the continuous glucose monitoring without the need for an enzyme.

Another interesting example of a wearable device enabled by 3D printing for sweat analysis was reported by Dias and coauthors (Dias et al., 2019). The 3D-printed device was fixed to the body of a volunteer and sweat was collected by a 3D-printed reservoir at which a flexible thermal-printed electrode was placed. The device was capable to monitor zinc ions in sweat by anodic stripping voltammetry using a bismuth-modified working electrode.

One important advantage of 3D printing technology in the development of wearable sensors is the fabrication of flexible devices with the electrochemical system integrated into the device. Hence, many possibilities can be envisioned for such applications.

### Other Electrochemical Cells and Electrodes

The DLP 3D printer was used to manufacture porous carbon electrodes used as anodes for microbial fuel cells (MFCs). The format that the MFCs were built increased significantly the metabolic activities of the microorganisms. The 3D printed anodes had better electrochemical results than those using carbon cloth anode and carbon fiber brush anode under the same conditions (Bian et al., 2018).

Spectroelectrochemical cells were built with ABS using an FFF 3D printer and were used to study the structural changes of the Prussian Blue at different voltage bias (dos Santos et al., 2019), as well as to perform voltammetry of the immobilized microparticles (da Silveira et al., 2021).

Like all equipment, 3D printers also have advantages and disadvantages, being the low cost, low time of production, and the use of little raw material pointed out as the main advantages. However, there are also disadvantages of the printed pieces such as limited thermal resistance and the relative difficulty in the operation of the machines. For example, the printing of electrochemical cells significantly improves the repeatability and reproducibility of the measurements, since they are manufactured specifically for each analysis or method, while this is a great advantage it is also a disadvantage, as this device is hardly used in a situation other than that for which it was designed.

In a recent review of the use of 3D printers to manufacture electrochemical detection systems, the authors cited publications that show how the researchers have increased the range of applications for 3D printers, for instance, to create equipment accessories in addition to electrochemical devices and substrates for these devices (Carrasco-Correa et al., 2021).

## Conclusion and Perspectives

In this review, we presented in an organized way the steps for the production of objects using 3D printing technologies and gave examples of how this technology is being used for the development of electrochemical devices. In this sense, several opportunities are open for the production of filaments and resins that can improve the printing process and increase the range of applications within chemistry, for instance, filaments that can withstand high temperatures without changing their physical, chemical, or mechanical properties. As mentioned at the beginning of this article, 3D printers work like small factories and these are modernizing very quickly, like closed loop 3D printer. These advances will allow the printing of entire electrochemical systems without the need for any other technology. Another possibility is the rapid manufacture of spare parts for old equipment, a recurring problem in universities in developing and underdeveloped countries. An application that is already a reality is the prototyping of cases and components for consoles, that is, 3D printers can even be used to make portable potentiostats, printing all the physical parts and many of their components.

## Abbreviations

ABS, Acrylonitrile butadiene styrene; AM, additive manufacturing; BIA, Batch-injection analysis; CAD, Computer aided design; CNC, Computer numeric control; DLP, Digital light processing, DMD, Digital micromirror device; DMLS, Poly jet direct metal laser sintering; EBM, Electron beam melting; FDA, Food and drug administration; FDM, Fused deposition modelling; FFF, Fused filament fabrication; FIA, Flow-injection analysis; HPLC, High performance liquid chromatography; MFCs, Microbial fuel cells; MJF, Multi jet fusion; MJM, Multi jet modelling; PEEK, Polyketones; PEI, Polyether imide; PEKK, Polyether ketone ketone; PETG, Polyethylene terephthalate glycol-modified; PLA, Polylactic Acid; PP, Polypropylene; SLA, Stereolithography; SLS, Selective laser sintering; TPE, Thermoplastic elastomer; TPU, Thermoplastic polyurethane; Tritan, Taulman tritan high tensile polyester; VP, Vat polymerization.
