# Supplementary material for: A 3D Printer Guide for the Development and Application of Electrochemical Cells and Devices
Source: Front Chem. 2021 Jul 2;9:684256. doi: 10.3389/fchem.2021.684256 (PMC8283263; doi:10.3389/fchem.2021.684256)
Supplement: Supplementary file 1 [file datasheet1.pdf]

Supplementary Information

A 3D printer guide for the development and application of electrochemical cells and devices

Ana Luisa Silva[1*], Gabriel Maia da Silva Salvador[1], Sílvia V. F. Castro[2], Nakédia M. F. Carvalho[1] and Rodrigo A. A. Munoz[2]

[1]Grupo de Catálise Ambiental e Sustentabilidade Energética, Instituto de Química, Departamento de Química Geral e Inorgânica, Universidade do Estado do Rio de Janeiro, Maracanã, 20550-013 Rio de Janeiro, RJ, Brasil.

[2]Núcleo de Pesquisa em Eletroanalítica, Instituto de Química, Universidade Federal de Uberlândia, 38400-902, Uberlândia, Minas Gerais, Brasil

*** Correspondence:**
Corresponding Author
analuisa_als21@hotmail.com

S.1 Software for 3D printers: examples of software used for modeling, slicing, modeling, and printing correction

There are countless CAD software types that cover all the steps of the 3D printing process, which starts with modeling. Some software programs stand out in this stage as SolidWorks, for example, which is one of the most employed modeling software. It is based on a parametric computation system, building 3D forms by geometric shapes. The software is excellent for 3D engineers and designers, being easy to use by beginner operators and enthusiasts too. CATIA software, on the other hand, supports multiple stages of product development, making it easy to collaborate between different subjects. There are possible modeling projects of electric, fluid, and electronic systems, for example, which makes the software useful to several industries (aviation, consumer goods, electronics, etc). Iventor software presents functional 3D projects and, in addition to modeling, it is possible to evaluate the mechanical behavior of the built pieces, simulating movements of the structure and external forces influences like gravity, for example. Besides them, there are other remarkable software programs, such as AutoCad, Fusion360, NX, Solid Edge, etc.

For the slicing step, PrusaSlicer presents itself as a new version of old famous slicing software, made by Prusa Research, a world reference 3D printer industry. It has some notable features, such as MSLA (resin) and multi-material support, smooth variable layer height, custom supports using modifier meshes, ability to "wipe" into infill, among others features. MatterControl is another example of slicing software; it is possible to slice using a variety of advanced settings support generation,

software bed leveling and with integrated controls for dual extrusion. Slic3r, Simplify3D, SliceCrafter also are examples of software.

Designed for processing and editing unstructured 3D meshes, Meshlab is an example of modeling correction software. It is used to edit, inspect, fix and repair STL files. This software is also used specifically in filling holes in meshes, depending on a good deal of technical knowledge to apply all the tools appropriately, being recommended to experienced users. Meshfix, however, is a simple alternative to STL repair. It can fix various defects in meshes, such as holes, non-manifold elements, and self-intersections. Autodesk Netfabb is one of the most famous software on the market. It presents STL repair tools with automatic, semi-automatic, and manual repair options, which allows the user to find the best solution for their project. Besides that, this software also covers the entire setup before 3D printing, being a robust and versatile tool. Autodesk Meshmixer, 3D Builder, and Blender are good examples of modeling correction software too.

There are several types of printing software, including the slicing ones cited before (PrusaSlicer, Slic3r and MatterControl, for example), which makes the project real. Repetier-Host, for example, is an open source and highly capable software of 3D printer control. It offers multi-extruder support, up to 16 extruders, multi-slicer support via plugins, and support for virtually any FFF on the market. This software also offers remote access via its server, being possible to access the 3D printer remotely. OctoPrint, in addition, presents a different way to control the printing jobs. Combined with a Wi-Fi-enabled device, this software allows control over the printer machine remotely via OctoPrint's web interface. The software accepts G-code of practically any slicing software and allows visualization of them before and during printing. Both are free, being great options for printing software.

S.2 Main printing parameters: important settings for slicing parts

To print a part in 3D, some steps need to be taken: the first step is modeling the object and these are saved in formats such as: .STL, .OBJ, .X3D, .AMF and others; the second step is the configuration of the printing/slicing parameters: to perform this part, files are modeled in a format other than .STL must be converted to .STL; thirdly, object slicing must be done, this process transforms the .STL file into a .G-code file, the only format that the printer understands. This .G-code file provides commands for the printer to perform all the necessary movements to build the object, such as: heating the nozzle, making automatic leveling, retracting or releasing filament, go up, to the side, turn off heating. The slicing works as a translator for the 3D printer and finally this .G-code file must be shared with the 3D printer via USB cable, memory card or command software, such as octoprint for it to start printing the object. We saw in the previous section that there are several modeling, slicing, printing, and modeling correction software. In this section, we will present the main parameters that need to be configured to obtain functional parts in FFF 3D printers: temperature, speed, type of support, filling, layer height, and flow.

Before using the printer for the first time, it is important that the build chamber is leveled and calibrated, this will prevent objects from being printed warped or from giving an error during printing. After choosing the filament, it is necessary to make the appropriate temperature adjustment for this material, using temperatures higher than the ideal for the chosen material can leave the pieces brittle, full of loose strings, and in severe cases, they can carbonize the material inside the extruder or nozzle causing clogging. Lower temperatures can cause low fluidity causing the lack of material in parts of the part, decreasing its mechanical resistance. To perform the temperature calibration for each filament, there are some features, such as printing a temperature calibration tower by adjusting the temperature

every 5 °C. There are several models of calibration towers in free repositories, for example, Thingiverse. Print speed is also an important parameter, because the higher the print speed, the lower the resolution of the parts. The printing speed must also be changed according to the size of the nozzle, because the larger the outlet diameter of the nozzle, the lower the printing speed must be so that more material accumulates inside the extruder, this causes the flow to maintain itself correctly and fluidic materials are put where you need it. When printing very large pieces, with many details or cylindrical, it is often necessary to print these over a support. The distance between the support and the printed layer needs to be carefully chosen so that the support does not join the piece, making the removal impossible. The size of the support needs to be adjusted so that it serves its purpose saving the use of the material in these regions, as they will be discarded. The height of the layer must also be adjusted according to the diameter of the nozzle, the lower the height of the layer the higher the resolution of the parts, this parameter also increases the printing time when choosing to make high resolution parts. The infill, wall and top/bottom are the most important parameters in relation to the resistance of the printed objects, where the choice of a piece with 100% filling will be denser, but it does not mean that it becomes more resistant. The more filled, the greater the filament expenditure and the printing time increases considerably. Tully and Meloni (2020) explain in detail these last parameters (Tully and Meloni, 2020).

S.3 Features of raw material: filaments

Although the PLA filament is indicated for visual prototyping, this filament has also been applied in surgical implants for bone fractures, autologous and heterologous grafts. There are studies on the use of PLA as material for medical sutures, extracellular matrix framework, and many others. (An et al., 2000; Rezwan et al., 2006; Ferreira et al., 2008; Lou et al., 2008). ABS is used in various applications, such as automotive parts, musical instruments, ATMs, helmets, luggage, toys, and many others. OBS: Prints of parts using PLA do not emit toxic fumes during printing like ABS.

Other filaments that are being used more extensively are the flexible filaments and although there are several types, PP and TPU that is a variation of TPE will be highlighted. The applications of these two filaments are varied, for instance, in the sealing of doors and windows, medical supplies, sealing of household appliances, toys, shoe soles, hinges, and others, representing good applicability in R&D.

An, Y. H., Woolf, S. K., and Friedman, R. J. (2000). Pre-clinical in vivo evaluation of orthopaedic bioabsorbable devices. *Biomaterials* 21, 2635–2652.

Ferreira, B. M. P., Steffen, A. M., Cardoso, T. P., Alberto-Rincon, M. do C., and Duek, E. A. R. (2008). Haste intramedular polimérica bioreabsorvível (PLLA/PHBV) para uso na recuperação de fraturas ósseas. *Polímeros* 18, 312–319. doi:10.1590/s0104-14282008000400010.

Lou, C. W., Yao, C. H., Chen, Y. S., Hsieh, T. C., Lin, J. H., and Hsing, W. H. (2008). Manufacturing and Properties of PLA Absorbable Surgical Suture. *Text. Res. J.* 78, 958–965. doi:10.1177/0040517507087856.

Rezwan, K., Chen, Q. Z., Blaker, J. J., and Boccaccini, A. R. (2006). Biodegradable and bioactive porous polymer/inorganic composite scaffolds for bone tissue engineering. *Biomaterials* 27, 3413–3431. doi:10.1016/j.biomaterials.2006.01.039.
